# Computational Modeling, Formal Analysis, and Tools for Systems Biology

**DOI:** 10.1371/journal.pcbi.1004591

**Published:** 2016-01-21

**Authors:** Ezio Bartocci, Pietro Lió

**Affiliations:** 1 Faculty of Informatics, Technische Universität Wien, Vienna, Austria; 2 Computer Laboratory, University of Cambridge, Cambridge, United Kingdom; University of Oxford, UNITED KINGDOM

## Abstract

As the amount of biological data in the public domain grows, so does the range of modeling and analysis techniques employed in systems biology. In recent years, a number of theoretical computer science developments have enabled modeling methodology to keep pace. The growing interest in systems biology in executable models and their analysis has necessitated the borrowing of terms and methods from computer science, such as formal analysis, model checking, static analysis, and runtime verification. Here, we discuss the most important and exciting computational methods and tools currently available to systems biologists. We believe that a deeper understanding of the concepts and theory highlighted in this review will produce better software practice, improved investigation of complex biological processes, and even new ideas and better feedback into computer science.

## Introduction

Computer science is currently central to a huge range of scientific areas. In its early days, its task was simply to translate a model expressed in a mathematical language into a computer program simulating that model. The field has progressed since then, yielding new domain-specific programming languages that are able to directly model a physical process.

In both cases, the computational implementation is perceived as a necessary methodological step for systems biologists, because the simple execution of a program provides an in silico numerical evaluation of hypotheses, avoiding the use of complex analytical methods and considerably reducing the costs of expensive in vivo or in vitro experiments.

Recently, the dichotomies between mathematical and computational models have also been subject to a debate (see also [1,2]) about whether or not the difference between them arises primarily from their ability to be directly executed [1] or from the different purposes and approaches adopted by scientists [2].

The novel concepts and principles (and well-designed tools) developed within the computer science community are accompanied by a domain-specific terminology (for example, executable models, expressivity, abstraction, model checking, reachability analysis, formal verification, and static analysis) that is scarcely known in other scientific communities such as systems biology. The introduction and assimilation of these concepts in fields other than computer science may back-propagate new ideas to computer scientists.

Recent works have discussed in detail how the methods borrowed from computer science have already benefited and can further benefit various problems in biology [1–6]. With respect to these previous research and review papers, our effort focuses on discussing how the use of newly developed tools could facilitate the understanding of the concepts, practice, and terminology acquisition in the current language of systems biologists. Furthermore, this review will take the further step of communicating the usefulness of using a temporal-logic framework for systems biologists who are looking beyond correlation toward event causality or patterns occurring in biological signals.

A nonexhaustive literature review, which is nonetheless an aid to understanding current progress in the field, is then presented.

## Computational Modeling

In the last decade, the area of systems biology has benefited greatly from computational models and techniques previously adopted only in computer science to assess the correctness and safety of a program. In this context, the design of a biological model becomes equivalent to developing a computer program. Various programming languages, often biological domain-specific, provide a means of describing the instruction sequence specifying the control flow of a biological process.

The syntax of the language defines the ways the symbols may be combined to create well-formed sentences or instructions. This specification is often represented in a textual way (i.e., a process calculus, rule-based system), but in several cases (i.e., Petri nets, statecharts, etc.), a graphical representation is also available. This helps the user to visualize the process with diagrams displaying the flow of the species in the reactions or the change in the internal states. The semantics reveals the meaning of the syntactically valid instructions by describing the behavior of the model and how it should be executed by the computer. It is also possible that a model specified using a particular language syntax may be executed using different language semantics: for example, a set of chemical reactions rules can be executed using a continuous semantics (ordinary differential equations [ODEs] on molecular concentrations) or a stochastic semantics (on the number of molecules), depending on the level of approximation and/or complexity [7] that we may want to achieve. For example, COPASI [8,9] is a tool for numerical simulation and analysis of biochemical networks for both their continuous and stochastic dynamics.

**Fig 1:**
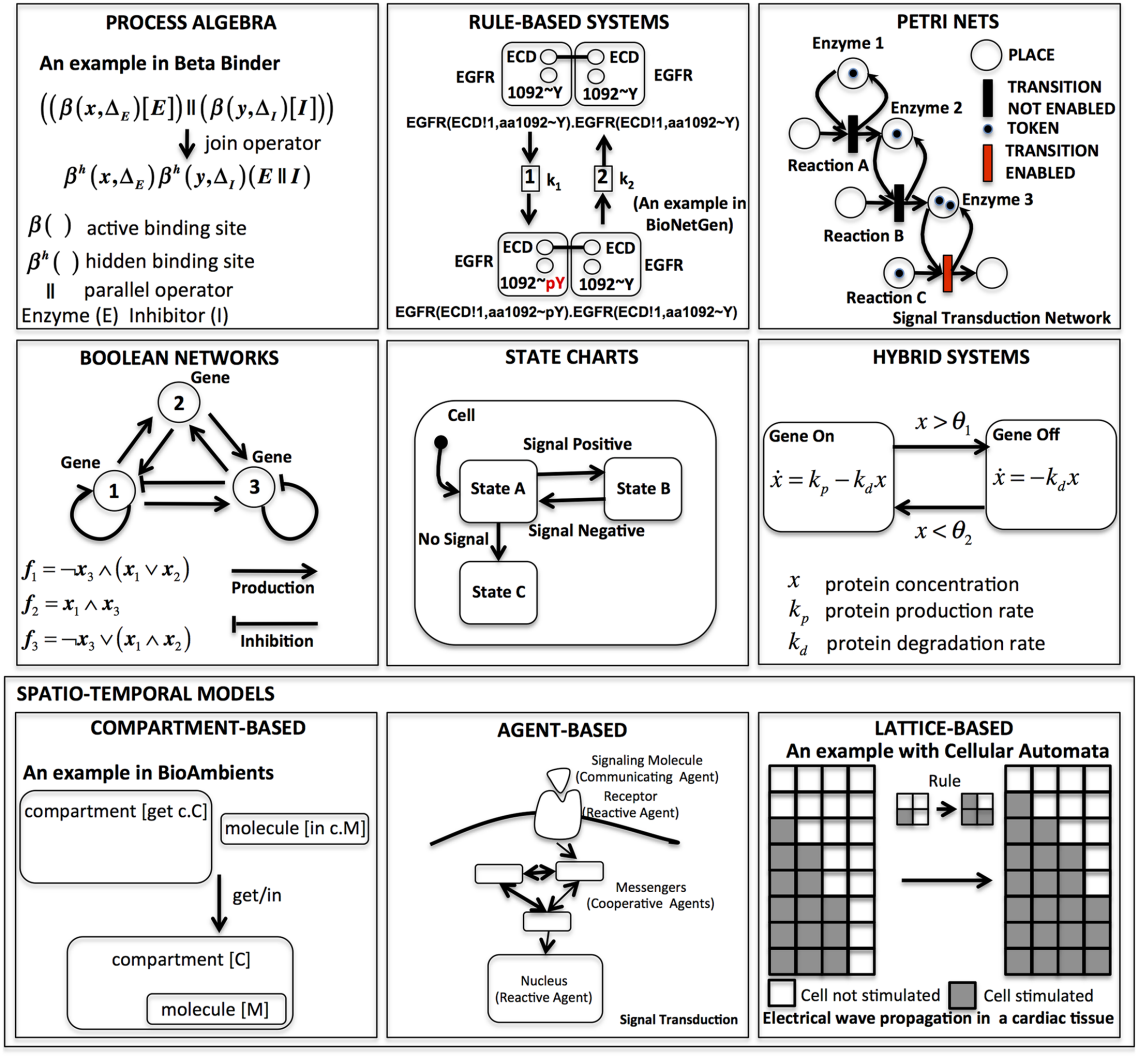
Most relevant examples of computational modeling approaches introduced with toy examples. Related tools are listed in Table 1. References for the examples are as follows: process algebras [12], compartment-based systems [21], rule-based systems [22], statecharts [23], hybrid systems [24], Boolean networks [25], Petri nets [26], agent-based models [27], lattice-based models [28].

In the following, we discuss the key features of the main computational modeling approaches that have fallen on fertile ground in systems biology. Fig 1 provides simple examples, inspired by case studies reported in the literature, of the modeling approaches considered.

### Process Algebras

In recent years, computer scientists have intensively investigated the use of process algebras (PAs) for the modeling and the analysis of biological systems [10–14]. The expressive power of PAs (see Fig 1: first row, first column) allows formal specification, without any ambiguity about the interactions, communications, and synchronizations between a collection of concurrent processes (also called agents). The reason for the interest in PAs for systems biology is that biological systems can be considered as concurrent reactive systems, where biological species can be modeled as processes interacting with each other. Another important feature of PAs in the modeling of complex (often multiscale) biological systems is their compositionality, which offers the possibility of defining the whole system, starting from the specification of its subcomponents. Furthermore, PAs usually permit formal reasoning about equivalences between processes. The leading examples of PAs in computational systems biology include Beta-Binders/BlenX [12], SPiM [15–17], Bio-PEPA [13], sCCP [18], and BioShape [19,20]. PA specifications are usually employed as intermediate models that are then executed or translated in other computational models using different semantics: continuous differential (ODEs), stochastic (Continuous Time Markov Chains), or abstract (transitions systems).

### Rule-based Systems

Rule-based modeling (see Fig 1: first row, second column) has gained a lot of attention among biologists, because its notation is very similar to the chemical reaction representation used in systems biology to model biochemical interactions between molecular species. Consider, for example, the classical enzymatic reaction where an enzyme (E) binds a substrate (S) and produces a product (P) by releasing the enzyme (E). This can be expressed in a very compact and concise description by using two simple rules:

1. E+S⇆ES
2. ES→E+P

One important feature of this modeling technique is that rules, unlike equations, are independent units, so they can be easily changed or modified. Furthermore, the simple syntax of rule-based models can be stored in a file as a human-readable text and can be edited and visualized using a graph representation. This makes rule-based modeling friendly for users without specialized mathematical or computer science skills. Rule-based models can then be translated, using different semantics, to generate other computational models in order to provide a quantitative (i.e., the amount of a species in time) [22,29] prediction or a qualitative (i.e., where time is abstracted away) understanding of the system’s emergent behavior. For these reasons, many rule-based modeling languages and tools, such as BIOCHAM [30,31], Kappa [32], BioNetGen [22,33], have become very popular among systems biologists in the recent years and have been intensively utilized in concrete case studies [34–36]. We refer to [3] for a more exhaustive review of rule-based modeling.

### Petri Nets

A Petri net (see Fig 1: first row, third column) is a directed graph whose vertices can be divided into two disjointed sets (bipartite graph), a set of nodes called “transitions” (meaning events that may occur, i.e., reactions), graphically represented by bars, and a set of nodes called “places” (meaning the conditions for a reaction to occur, such as the presence of a molecule), graphically represented by circles. Arrows interconnect these nodes, showing the direction of flow, with this main rule: a place node can be connected only to a transition node and vice versa. The data (i.e., species) are generally represented as “tokens”, signified by black marks. The tokens are consumed from the input places through the transitions and then created in the output places. A transition “fires” whenever it is enabled by the presence of some tokens in one of the places directly connected to it. A concurrent semantics specifies the evolution in time of the token distribution. This modeling framework was introduced by Carl Adam Petri in 1962 with the purpose of describing chemical processes [37], but then was also intensively employed in computer science to specify and analyze concurrent and distributed systems. It is not surprising that this intuitive and graphical modeling style is popular among computational systems biologists [26,38–40] to describe biochemical reaction systems, where the tokens are interpreted as single molecules of the species involved. The Petri net formalism, as shown also in [41], provides a natural framework in which both qualitative (given by the static structural topology of the Petri nets) and quantitative (given by the time evolution of the token distribution) analysis are tightly integrated. Important tools for Petri nets used in computational biology are Snoopy [26], MARCIE [42], GreatSPN [43,44], and Pathway Logic Assistant [45,46].

### Boolean/Qualitative Networks

Boolean networks (see Fig 1: second row, first column) were first introduced by Kauffman [25] and then by Thomas [47,48]. They are often used to approximate the dynamics of genetic regulatory networks by considering genes either activated (true state) or deactivated (false state). A Boolean network is defined in terms of Boolean variables, each one updated by a Boolean function that determines the next truth value state given the inputs from a subset of those variables. This modeling technique, even though it usually introduces a coarse approximation by neglecting intermediate states, is widely employed to analyze the robustness and stability of genetic regulatory networks. For instance, by generating initial random configurations, it is possible, by executing this model, to detect singleton attractors (also called fixed points), where the system is stable. Relevant tools for Boolean networks analysis in systems biology are GINsim [49–51], BoolNet [52], and BNS [53,54]. Qualitative networks, introduced recently in [55], extend the Boolean network, allowing its elements to assume a finite number of possible values. This feature provides biologists with more flexibility than just Boolean values and enhances the variety of behaviors that it is possible to model with this formalism. The tool for modeling and analysis of qualitative networks is Bio Model Analyzer (BMA) [56].

### Statecharts

Another natural way to model the dynamics of a biological system is to specify the sequence of the states characterizing its behavior [23]. For example, when a phosphate group is added to some proteins, their functional behavior can change to a phosphorylated state, enabling other potential protein—protein interactions. A system remains in a state until the occurrence of some event (e.g., the activation or inhibition of a gene) moves its internal behavior from one state to another. This characteristic makes a biological system a multiscale reactive system in which event-driven concurrent interactions, occurring at different levels (molecular, cellular, tissue, organ, or population level) or between levels and with different timing and order, determine its emergent behavior. The statecharts notation (see Fig 1: second row, second column) is then a suitable formalism to present, in a graphical representation, the interdependence among the states of a reactive system. Several slightly different versions of these state diagrams have been proposed with different semantics.

The statecharts introduced by Harel [57] have been the most popular among biologists because they offer appropriate constructs (hierarchy of states with transitions, events, conditions, orthogonal regions, etc.) to handle the complexity of modeling biological systems. The classic statecharts notation, in fact, would require one to specify any possible combination of parameters as a distinct state, leading to an explosion of the number of states. Among the tools for statecharts, the most relevant in systems biology is IBM Rational Rhapsody [58,59].

### Hybrid Systems

Hybrid systems [60] (see Fig 1: second row, third column) extend the state-based discrete representation previously mentioned with a continuous dynamics (generally ODEs) in each state (or mode). Hybrid modeling techniques [24] are gaining more and more attention in systems biology [61] for their ability to capture the behavior of biological systems that exhibit clear switching characteristics. In particular, sigmoidal switches occur everywhere in biological models: molecular (an example is the sigmoidal behavior exhibited by Hill-type kinetics), cellular, tissue, organ, and population models. Hybrid modeling is generally suitable to combine qualitative (given by the discrete state) and quantitative (given by the continuous dynamics) information [62]. In the last decade, several hybrid system identification (hybridization) methods have been proposed in the literature [63–66] to approximate complex nonlinear dynamics with piecewise-linear [67,68] or piecewise-multi-affine functions [66,69–71], making such models amenable to formal analysis [60,66–71] and improving large-scale simulation of multicellular ensembles [72–74]. It is noteworthy that widely used mathematical platforms, such as Matlab [75] and Simulink [76,77], enable the user to model and simulate hybrid systems. Other relevant tools for hybrid systems modeling in biology are Rovergene [71], BioDivine [69,78,79], Breach [80,81], dReach [82,83], and S-TaLiRo [84].

### Spatio-temporal Models

Continuous state deterministic spatiotemporal systems (see Fig 1: third row) are generally formulated in terms of “reaction-diffusion” systems taking the form of semilinear parabolic partial differential equations (PDE). In the discrete state setting, compartment-based models (i.e., membrane computing), agent-based models, and lattice-based computational models (i.e., cellular automata, cellular Potts) have been employed to simulate the collective behavior of cellular structures. All of these models display a wide range of behaviors emerging from local and nonlocal interactions such as traveling waves (i.e., cardiac tissue) [85], Turing patterning [86,87], and spirals [88,89].

#### Compartment-based models

Biological systems are generally organized in compartments (i.e., cell membrane, cell nucleus, organelle), exchanging molecules between them according to certain rules. Compartment-based models (see Fig 1: third row, first column) are specialized to capture several biological characteristics, such as the dynamic rearrangements of the compartments (a typical behavior observed in the mitochondria) and the transport of molecules between them.

The study of the membranes separating the compartments has also initiated a new area within computer science called membrane computing, which aims to discover new bio-inspired computational paradigms, such as the P Systems [90]. However, these models are more suitable for the theory of computation than for modeling in systems biology.

Another relevant modeling framework is BioAmbients [91], a process algebra enriched with special operators able to specify merging, splitting, and communication between compartments. BAM [92,93] is a tool for executing stochastic BioAmbients. BioAmbients evolved into Brane Calculus [94], which offers a specially designed language to describe the dynamic behavior of membranes. Whereas, in BioAmbients, the ambient (i.e., compartment) plays an active role in dictating which processes may enter or exit from it, Brane Calculus offers a different perspective, in which the membranes have the control and play the role of coordinators. To the best of our knowledge, there is not yet an implementation available for it.

#### Agent-based models

Agent-based models [95,96] (see Fig 1: third row, second column) consider a collection of autonomous decision-making entities, called agents, which individually sense the environment and make decisions on the basis of a set of rules. Although, at the simplest level, an agent-based model consists of a system of agents and the relationships between them, it can still exhibit complex behavior patterns in terms of changes and adaptation in response to environmental challenges or to neighboring agent behaviors (for example, competition or collaboration).

Because all individuals in a population are explicitly represented, they can have unique histories and behaviors. More complex agent-based models sometimes incorporate sophisticated learning and adaptation rules based on neural networks, evolutionary algorithms, or other techniques. The single-cell-based models represent one of the most promising aspects, in which agents have many cellular functional and structural features and behavior, inching toward reality and enabling the detection of phenomena at different intermediate scales of biosystems.

Cell-based models can express important behavioral characteristics of a cell, such as the dynamics of its replication and information on each stage of its development (i.e., cell geometry, size, and mechanical properties).

A single-cell-based model should be able to understand how stage-dependent cell—cell interactions at microscopic scale will lead to cell—tissue interactions and stage heterogeneity at mesoscopic level and mechanical properties of the tissue at macroscopic level. Models could be implemented using FLAME [27,97] and REPAST [28,98], for example.

#### Lattice-based models

A lattice (see Fig 1: third row, third column), which defines a regular repeated graph, formed by identical n-dimensional closed grid sites and characterized by periodic or fixed boundary conditions in each direction, is particularly suited for systems description of interconnected processes at the molecular, cellular, and the tissue or organ level. These natural levels can approximately be connected to a microscopic (molecule motion and interactions), mesoscopic (cell division and motion, cell—cell interactions, cell-matrix), and macroscopic scale (tissue and organ mechanical properties), respectively.

Cellular automata [99] are discrete dynamic systems—discrete in space, time, and state. Cellular pattern formation can be seen as arising from short-range (such as adhesive forces and cell—cell signaling) and long-range (such as mechanical stress fields or diffusing chemicals) interactions. A Bethe lattice (or Cayley tree) [100] is a hierarchically ordered, cycle-free network without ends that has been applied to immunological (idiotypic) networks.

In multiscale lattice-based models, we can observe what happens at almost all scales, from the whole organism down to the molecular level; however, putting things together in order to obtain real understanding is much more difficult and involves scaling up and homogenization of models across multiple spatial scales and related asymptotic techniques for the analysis of multiple time scales. This problem could be overcome by using energetic considerations, such as in the cellular Potts model (also termed the Glazier Graner-Hogeweg model), which are based on the stochastic Monte Carlo method on a regular lattice [101,102]. The objects, either discrete generalized cells (unicellular organisms, clusters of cells, individual cells) or continuous fields (such as gradients of nutrients or small molecules), are associated with an energy description of processes such as cell—cell adhesion or cell—nutrient interaction. Lattice rearrangements, which simulate the evolution of the system, are driven by the energy minimization of a Hamiltonian function.

A very general and flexible framework for Potts model development is CompuCell3D [103,104], which has been used to model a variety of anatomical and pathological conditions at cell, tissue, and organ levels. This framework succeeds in combining both a rigorous energetic and mechanical treatment of the process with an intuitive and insightful biological description. There is growing interest in network ensembles approaches. Multilayer networks and, in particular, multiplex networks (in which different networks share the same nodes) could be analyzed using network entropies to evaluate and quantify the correlations between interdependent networks. For example, in biological systems, gene, protein, and metabolite networks have strong correlations and interdependencies that cannot be fully pictured in terms of single graphs [105].

## Formal Analysis

The modeling languages presented in the previous section play a key role in supporting the rigorous specification of the mechanisms observed experimentally, helping scientists in the formulation of new hypotheses. Once a model is constructed, a suitable tool can parse the syntax of its specification and interpret it according to the semantics of the chosen modeling language. A model can also undergo a process of compilation that automatically translates it into a computer program simulating the biological process under investigation. The generated program can be used to predict the emergent behavior of a system with certain initial conditions. This contributes to the testing procedure and to reducing the number of costly experiments, concentrating all efforts and resources only on those that promise to reveal novel, interesting mechanisms.

Another advantage is the possibility of inheriting several methods and tools that are commonly developed and employed within the computer-aided verification community to formally check the correctness of a program’s behavior. In the context of systems biology, these methods are becoming very useful for reasoning and analyzing models, validating new experimental results, automatically checking behaviors of interest, and identifying the inputs or parameters of the system enforcing a desired behavior.

The formal verification of a program consists of proving that its execution satisfies a given specification of the possible behaviors it should display. In the following, we will first present some logic-based languages used to specify temporal behavioral properties rigorously and concisely.

These languages, first developed within computer science, are now also employed in several case studies of systems biology to model recurrent patterns in biological signals or simply the order of relevant biological events. We will then discuss three well-established formal verification techniques designed first to verify programs and now widely employed to analyze biological models: model checking [6,70,71,106–110], runtime verification [80,111–116], and static analysis [93,117–119].

### Temporal Logics

**Fig 2:**
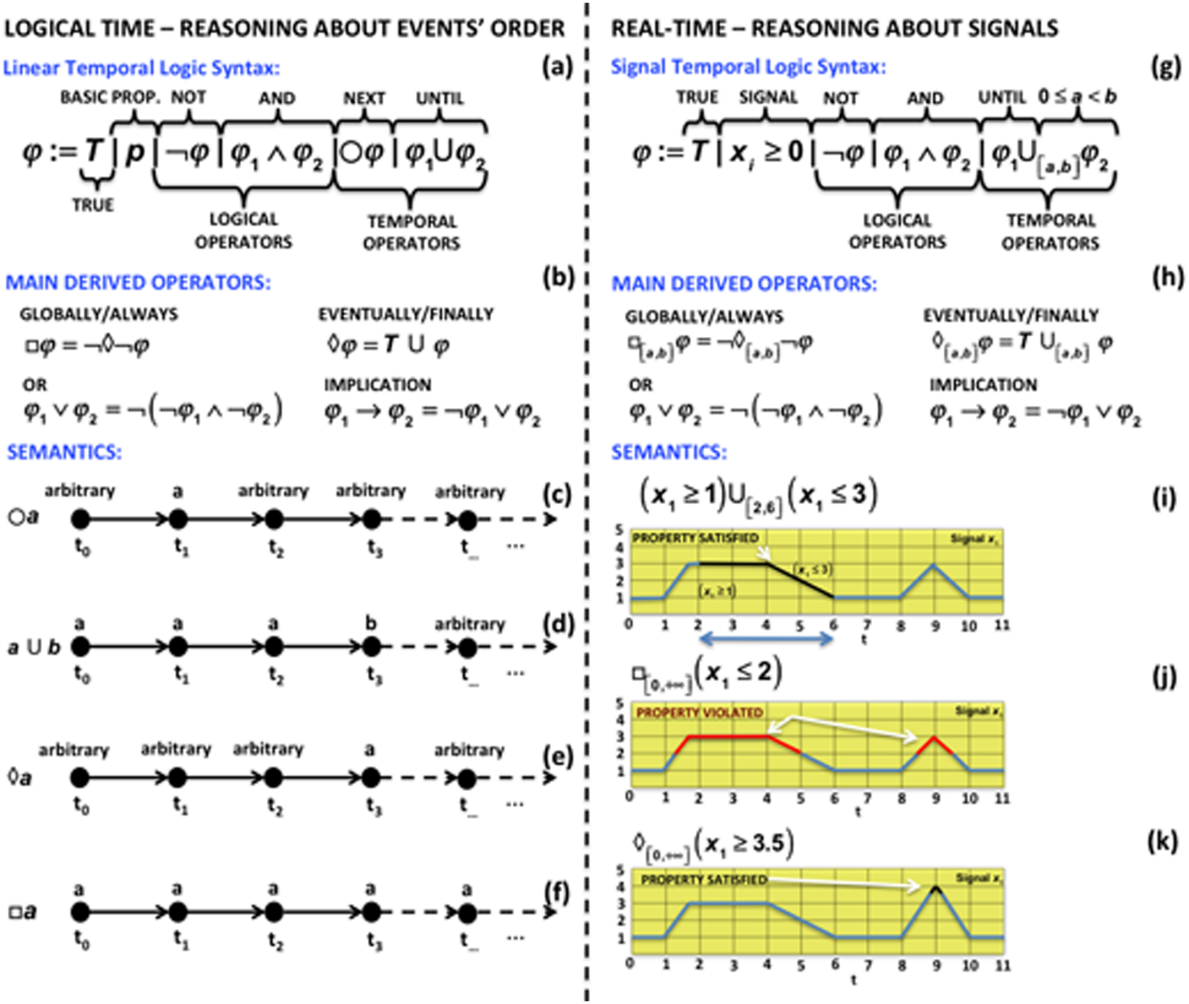
Examples of temporal logics. Comparison between the main features of the LTL (left) and Signal Temporal Logic (STL) (right) in terms of syntax (top), operators (middle), and semantics (bottom); the black circles represents a propositional state, and the arrows represent the next step in time.

Temporal logics [120–123] are very concise languages for rigorously specifying the occurrence of specific temporal behaviors. One of the most popular temporal logics is Linear Temporal Logic (LTL), introduced by Pnueli in 1977 [120] to reason about the order of events occurring during the execution of a program. The LTL syntax is given by the grammar shown in Fig 2a. The basic proposition *p* indicates a Boolean value that may express the relationship between a state variable of the system and a value for a particular time instant. For example, we can specify that the concentration of the specie *x*_1_ is greater than or equal to a certain threshold *r* (*x*_1_ ≥ *r*) or that a specific biological event *e* (e.g., phosphorylation) should occur. More complex logical formulas can be obtained by combining propositions using logical operators such as or (∨) and not (¬). The other classical logical operators such as and (∧) and implication (→) can be derived by combining the previous two, as shown in Fig 2b.

The LTL syntax also includes two temporal operators: the next (○) operator, which means that a formula *φ* should hold in the next step (see Fig 2c), and the until (U) operator, which requires a formula *φ*_1_ to hold until a formula *φ*_2_ becomes true (see Fig 2d).

From the until operator, it is possible to derive other very suitable temporal operators: the eventually (⋄) operator specifies that a formula *φ* will finally become true at some point (see Fig 2e), and the always (□) operator states that a formula *φ* should remain true forever (see Fig 2f). The combination and the nesting of the basic propositions with the logical and temporal operators allow the specification of several different types of temporal behaviors.

The most common temporal patterns are:

1. Reachability properties, in which an event will finally happen. For example, we can express the property "the event of protein A production (event *A*_↑_) will finally occur" with the LTL formula *φ* = ⋄*A*_↑_. This specification does not guarantee that the same event will happen again after it has occurred.
2. Liveness properties, in which an event will always finally happen. This specification guarantees that the same event will also happen again after it has occurred. For example, the property "always the event of the degradation of protein A (event *A*_↓_) implies eventually the activation of gene B (event *B*_↑_)" [124] can be specified with the LTL formula *φ* = □(*A*_↓_→ ⋄*B*_↑_).
3. Safety/invariant properties, in which a system will always satisfy a certain requirement. For example, the property "the number of the osteoclasts, the cells degrading/digesting the bone matrix, *x*_*oc*_ during bone remodeling will be always less than a particular concentration *c*" [107] can be specified with the LTL formula *φ* = □*p* with *p* = (*x*_*oc*_ ≤*c*).
4. Stability properties, special cases of liveness properties, in which eventually an invariant property will hold. For example, the property "finally the skin cell proliferation *x*_*c*_ will reach a stable level *l*" [125] can be expressed with the LTL formula *φ* = ⋄□*p* with *p* = |*x*_*c*_ −*l*|≤*ε*.
5. Oscillatory properties. For example, the property "the concentration of a protein *x*_*p*_ is oscillating between two levels *t*_*a*_, *t*_*b*_ with *t*_*a*_ < *t*_*b*_" can be written as the LTL formula *φ* = □((*p*_1_ →⋄*p*_2_) ∧ (*p*_2_ →⋄*p*_1_), with *p*_1_ = *x*_*p*_ ≤ *t*_*a*_ and *p*_2_ = *x*_*p*_ ≥*t*_*b*_

LTL operates on a single path of the model execution, and a temporal property can be formulated only for one possible trajectory of the system. Other temporal logics such as computational tree logic (CTL) [126] and CTL* [127] have in their syntax special quantifiers that enable the specification of properties over all the possible trajectories or branches in time: universal quantifier (∀) specifies that a nested formula should be true for all the possible trajectories, while the existential quantifier (∃) requires the formula to hold in at least one of the possible trajectories.

All the aforementioned temporal logics consider only the temporal order of the events and not the actual time at which they really occur. For example, it is not possible to specify that a formula should hold after two units of time and before three and a half units of time. Even if we decide to discretize the time, recording all the events at each time step, the syntax of these logics is not equipped to deal directly with the specification of real-time intervals.

Real-time temporal logics [128–131] overcome these limits by using a continuous time semantics and embedding a time interval in the until temporal operator.

The Signal Temporal Logic (STL) [131,132] is an example of a real-time temporal logic suitable for many biological case studies [115,116,133–135]. STL extends LTL with the continuous time semantics and with predicates over real variables (see Fig 2g and 2h).

Fig 2i shows how the until operator of the STL syntax is enriched with the possibility of specifying a continuous time interval [*a*, *b*] within which the first formula *φ*_1_ should hold until *φ*_2_ holds. STL operates on a continuous piecewise representation of a sampled signal. As illustrated in Fig 2j and 2k, the STL semantics uses interpolation to determine the point between two samples where the formula will start to hold or to be violated.

STL has two possible semantics: a qualitative semantics returning a yes/no answer to the question of whether the system satisfies or violates the specification, and a quantitative semantics also providing a measure of robustness [111,113] of how much the system violates or satisfies the specification. Negative robustness implies property violation, while positive robustness implies property satisfaction. As we discuss later in this section, this value can be used to guide the parameter synthesis of a biological model with unknown parameters.

### Model Checking

Model checking is an automatic formal verification technique able to check the emergence of a particular behavior in a biological model. This technique operates over a discrete time model with a finite number of states, called a Kripke structure [136], where the execution of a model triggers a sequence of events determining the truth value of the propositions of a temporal logic formula. A Kripke structure is a special labeled graph in which the nodes represent the reachable states generated by executing the biological model, and the edges represent the state transitions. A labeling function maps each node to the set of propositions that hold in the corresponding reachable state. A transition relation specifies the set of possible successors for each state.

Each node always has a successor or a loop transition starting and ending in the same state, representing nonterminating computations where the evaluation of the atomic propositions does not change (also called fixed point). Suitable user-friendly tools can translate the biological models specified with one of the formalisms presented in the previous section into a Kripke structure representation that can be analyzed with very efficient model checkers such as NuSMV [137,138] or CADP [139]. The main drawback of this technique is that the number of states of a model usually grows exponentially in the number of its parameters, giving rise to the state explosion problem. In order to tackle this, the majority of model checkers do not explicitly represent the states, but represent sets of states symbolically [140,141]. For example, the states and the transition relation of a Kripke structure can be encoded as a binary decision diagram (BDD) [140], a very compact acyclic graph data structure used to represent a Boolean function as well as sets or relations in general. The logical operations required by model checking are then interpreted as operations over sets and implemented by polynomial-time graph manipulation algorithms [140] directly on this representation of sets. The works of Bryant [140] on BDDs and of McMillan [141] on symbolic model checking provide more details on the symbolic approach.

Model checking techniques have also been extended to many other computational models that can be regarded as Kripke structures, such as continuous- and discrete-time Markov chains (CTMC and DTMC) (by adding probabilities) [6], Petri nets [41], hybrid systems (by adding continuous dynamics) [142], and spatial lattice-based models (using the quad-tree representation) [88]. In the case of CTMC and DTMC, the analysis may benefit from using probabilistic model checkers such as PRISM [6,143,144], which provide a real number in the interval of [0,1] corresponding to the probability that the system model will satisfy the property of interest. The algorithm used to calculate this probability can return either the exact solution [123], if it operates directly on the structure of the Markov chains, or an approximated solution, when it measures statistically [145] the probability of satisfying a property for a set of samples, generated using a Monte Carlo simulation of the system model. This statistical approach can be applied not only to the classical DTMC and CTMC models, but also to stochastic hybrid systems [146], in which the continuous dynamics are calculated by integration and the discrete transitions are chosen nondeterministically by following a probability distribution.

### Runtime Verification/Monitoring

Another way to overcome the state explosion problem of model checking is to focus the analysis on a single execution trace instead of performing an exhaustive verification. Runtime verification is a lightweight yet powerful verification technique that aims to check whether the current execution of a program (i.e., the time series of the concentration of the protein expression during a gene regulatory network simulation) satisfies or violates a property of interest.

The emergent property is still specified in terms of a LTL formula or one of its extensions, but in this case only a single behavior is evaluated. Monitoring does not require a system model but only a set of observable, discrete, or continuous signals that can be collected during a wet-lab experiment or generated by numerical simulation. As previously mentioned, in the last decade, LTL has been extended to specify properties of real-valued variables defined over dense real time. A pioneering example of LTL with predicates expressed in terms of constraints over reals is LTL(ℝ), presented in [147,148], and then implemented to monitor numerical simulations of biological models in BIOCHAM [108].

In addition, STL [131] extends LTL with a continuous time semantics and with predicates over real variables and is implemented in the Breach [80] and S-TaLiRo [84,149] tools. In these tools, the evaluation of the STL formula robustness for a particular trajectory through monitoring is used in combination with sensitivity-based analysis techniques [107,114,115,135] or stochastic-based optimization techniques [116,149] to steer the simulation of a biological model toward the parameter regions in which it would display the property of interest.

### Static Analysis

As the term “static" indicates, this analysis is performed on the static description of the model without actually executing it. The principles of static analysis originated in the field of compiler optimization. Nowadays, this approach is widely employed in software verification, its key role being to detect potentially vulnerable code in safety-critical applications.

While model checking generally needs to explore all the states originated by executing the semantics of the model, static analysis operates on the syntactic level of the specification or by using abstract interpretation [150] over finite approximations of the possible model executions [151]. Static analysis can reveal important information regarding the model specification (e.g., the control structure, the flow of species concentrations, the interactions among species), without performing all of the underlying concrete calculations. In the last decade [117], static analysis has also become a useful technique to analyze biological models [93,118,119]. In [93], the authors successfully employ this technique to analyze biological pathways. Given a formal model in BioAmbients of the LDL degradation pathway, the authors compute a fine over-approximation of the possibly infinite reaction sequences that the model specifies. This approximation is “safe”, meaning that all the reaction sequences that do not appear in the analysis are not possible.

Static analysis is crucial for dealing with the complexity of real systems (see also [118,119] for other important examples in systems biology), in which model checking all the reaction sequences will fail, owing to the state explosion problem. However, it is not as precise as executing the model: it is not possible to guarantee that all the reachable states in the over-approximation are also reachable in the original model’s behavior.

In some cases (see, for example, the Rovergene tool [71]), static analysis and model checking techniques are combined. The first constructs an abstract domain using suitable abstractions. The second provides a logical framework to search in the abstract domain if a set of states is not reachable. This guarantees that they will never occur in the original model’s behavior. Examples of this analytic approach can be found in several case studies: genetic networks [71], loss of cardiac cell excitability [66], and bone remodeling [107]. Static analysis has also been used in [152] to relate different semantics and formalisms used for describing reaction systems.

## Tools

We now use the concepts previously discussed as a guide to choosing among the several tools available.

**Fig 3:**
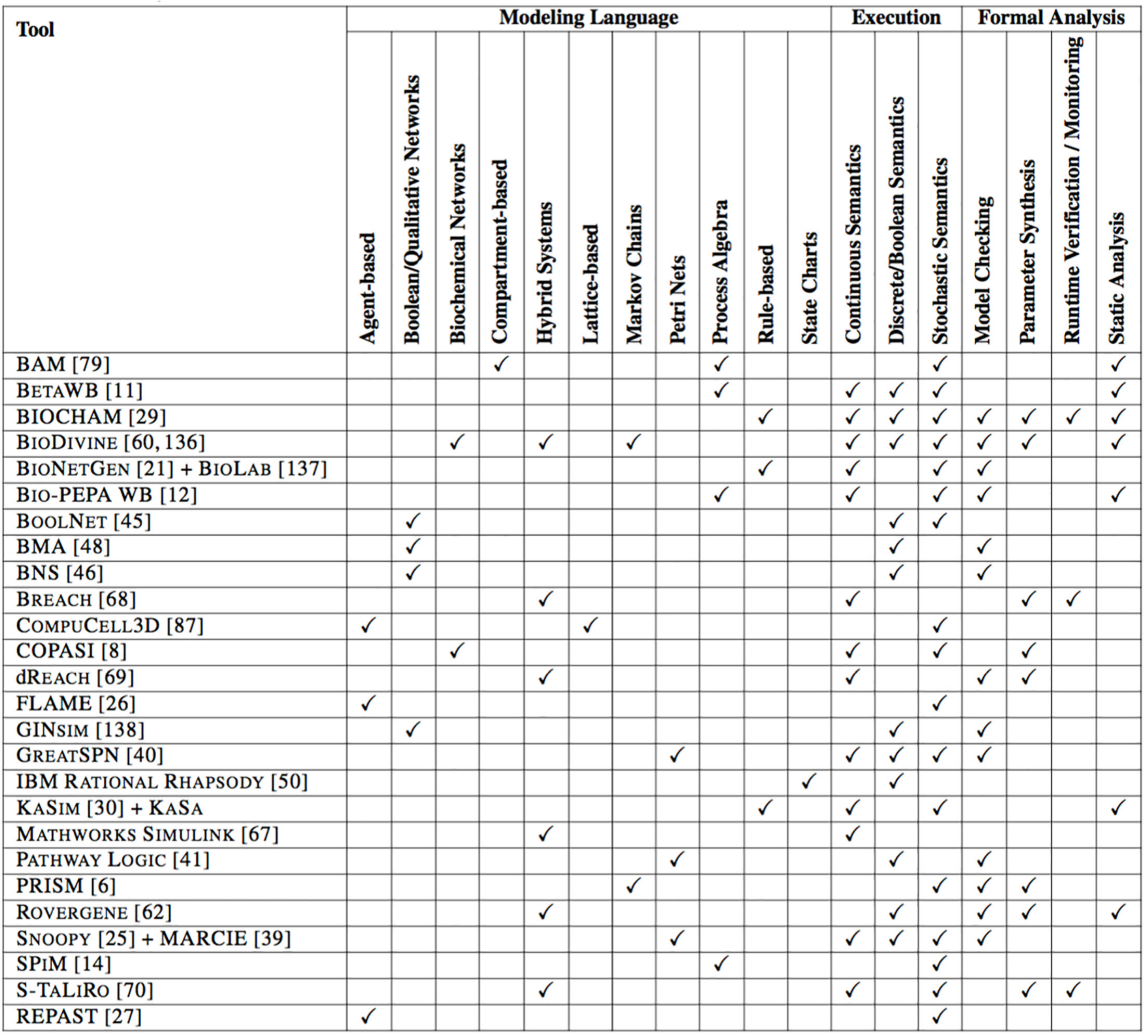
Summary of the features for the selected tools. Tools are classified by the supported computational modeling language, their execution semantics, and the formal analysis that can be performed, based on the literature.

Fig 3 and Table 1, though not purporting to be complete, present a selection of software closely related to the topics discussed in this review. In Fig 3, we classify the listed tools by the computational modeling language, the supported semantics of execution, and the formal analysis that can be performed, based on the literature. We also specify if the tool supports a mechanism to tune the model’s parameters, guided by the formal analysis.

**Table 1:** Summary of the main case studies in systems biology for the listed tools.

Tool	Main case studies
BAM [92]	LDL degradation pathway [93]
BetaWB [12]	The MAPK biochemical pathway [12], cell-cycle [11]
BIOCHAM [30]	Mammalian cell cycle control [34], G protein-coupled receptor kinases [31]
BioDivine [69,78]	Genetic regulatory networks [79]
BioNetGen [22] + BioLab [33]	HMGB1 signal pathway [35] Analysis of T-cell receptor signaling pathway [33]
Bio-PEPA WB [13]	Plant circadian clock [14]
BoolNet [52]	Genetic networks [52]
BMA [56]	Biological signaling networks [55]
BNS [53]	Cell cycle sequence of fission yeast [54]
Breach [80]	Collagen proteolysis [115], Cellular iron homeostasis network [81]
CompuCell3D [103]	Vertebrate segmentation and somite formation [104]
COPASI [8]	Biochemical networks [9]
dReach [82]	Cardiac cell hybrid models [83]
FLAME [27]	Sperm behavior [97]
GINsim [49]	Diversity and plasticity of Th cell types [50]
	MAPK network on cancer cell fate decision [51]
GreatSPN [43]	Signal transduction pathways for angiogenesis [44]
IBM Rational Rhapsody [58]	T-cell activation with statecharts [59]
KaSim [32]	EGFR signaling [36]
Mathworks Simulink [76]	Heart model for pacemaker verification [77]
Pathway Logic [45]	Sporulation initiation in *B*. *subtilis* [46] MAPK signaling network [46] EGF stimulation network [45]
PRISM [6]	Biological signaling pathways [6,143,144], bone pathologies [107]
Rovergene [71]	Synthetic transcription cascade [71], myocyte excitability [66], bone remodeling [107]
Snoopy [26] + MARCIE [42]	Systems and synthetic biology [41]
SPiM [15]	Modeling of the EGFR network [16], MHC class I peptide optimization [17]
S-TaLiRo [84]	Modeling of the insulin-glucose regulatory system [149]
REPAST [28]	Bone remodeling [98]

While each modeling language was developed to solve a real problem, different modeling languages may map into the same program. Knowledge of the syntax is needed in order to carry out static analysis. The executable program, on the other hand, is no longer syntax-dependent. Quantitative analysis (i.e., simulation), which considers the time dimension, is then performed on the output produced by the program.

In the second column of Table 1 (main case studies), references to some applications are presented. The large variety of tools will accommodate the current rich interdisciplinary and multidisciplinary systems biology scenarios. Scientists with different backgrounds may have different initial preferences and later move in various directions, generating the conditions for extensive exchange of ideas and methodological innovations.

## Conclusions and Vision

The growing availability of large amounts of data (i.e., big data) will allow models to be tested very finely. Spatial data could be collected in three dimensions (thanks, perhaps, to microscope imaging advances), capturing the formation of patterns, niches, molecular associations, and multiscale features. The time dimension could range from molecular events (for example, DNA mutations or epigenetic changes) to organism development, circadian, species evolution, and other meaningful periodicities.

The development of new, efficient tools will motivate others to generate new computational models or to improve the existing ones. This will increase the community of scientists sharing their knowledge through standardized computational models reproducing numerically the behavior of the biological process under investigation. With computational modeling acquiring better capacity to describe biological systems and processes at a level useful for prediction and to suggest experiments, it will trigger a useful feed-forward process with experimental biologists.

The tools described in this paper can already accommodate different complexly structured properties of biological processes and could be used separately or in different combinations and architectures. This will enable biologists to answer complex questions. For example, temporal logics, in particular, will have a profound impact in systems biology by helping to transform cause—effect relationships into objects that can be manipulated both mathematically and computationally. In epistatic control, temporal logics can be used to model two or more causal factors as interacting mechanistically with respect to the observed phenomenon. Doing so will establish powerful connections, with reasoning based on logic and statistics and the mechanisms and processes that underlie the observed behavior.

One future interesting research direction that we envision is the extension of the current formal analysis techniques and temporal logics to the spatial domain. For example, understanding how a spatial pattern emerges from the biochemical level acting at the cellular level (i.e., morphogenesis in developmental biology) is currently very challenging because of both the high computational complexity required by the spatiotemporal modeling and the lack of a suitable specification language to specify the spatiotemporal patterns of interest [86,87,153].

Furthermore, the rapid progress of modern technologies for healthcare has led to a new generation of devices called medical cyber-physical systems [154], in which smart and collaborative computational elements control the biological systems. Examples include pacemakers, biocompatible and implantable devices, insulin pumps, electro-anatomical mapping and intervention, robotic prosthetics, and neurostimulators. Here, the computational modeling of the biological part is indispensable to the development of efficient and safe controlling devices. Furthermore, the successful application of formal analysis techniques and tools to verify the correct and safe behavior of these systems will have an economic impact on our society by reducing warranty, liability, and certification costs. We believe that the concepts and the computational tools described here represent core elements of computational description, particularly in the framework of systems biology, and will have some relevance to both newcomers and experts.
